# Theoretical Investigation of Elastic, Electronic, and Thermodynamic Properties of Half-Heusler Semiconductors ZrNiPb and ZrPdPb Under Pressure

**DOI:** 10.3390/nano15030241

**Published:** 2025-02-05

**Authors:** Xiaorui Chen, Xin Zhang, Zhibin Shao, Jianzhi Gao, Minghu Pan

**Affiliations:** 1 School of Mechanical and Material Engineering, Xi’an University, Xi’an 710065, China; 2 School of Physics and Information Technology, Shaanxi Normal University, Xi’an 710119, China; x.zhang@snnu.edu.cn (X.Z.); jianzhigao@snnu.edu.cn (J.G.); 3 Physics Laboratory, Industrial Training Center, Shenzhen Polytechnic University, Shenzhen 518055, China; zhibin_shao@szpt.edu.cn

**Keywords:** elastic properties, electronic structures, thermodynamic properties, half-Heusler semiconductor, first-principles calculations

## Abstract

The half-Heusler semiconductors ZrNiPb and ZrPdPb have attracted considerable attention due to their excellent thermoelectric performance, owing largely to their appropriate energy bandgap. However, the bandgap is sensitive to pressure, which may influence their thermoelectric behavior. In this study, the effects of pressure on the elastic, electronic, and thermodynamic properties of the half-Heusler semiconductors ZrNiPb and ZrPdPb are investigated based on first-principles calculations combined with the quasi-harmonic Debye model. After verifying their structural, dynamic, and mechanical stability, we found a small indirect bandgap of 0.36 eV for ZrNiPb and 0.49 eV for ZrPdPb, and they increase with increasing pressure. According to the obtained elastic modulus, ZrNiPb and ZrPdPb become more and more ductile as the pressure increases. In addition, the thermodynamic properties of ZrNiPb and ZrPdPb are investigated using the quasi-harmonic Debye model, as implemented in the Gibbs program, which will provide a reference for the experiment.

## 1. Introduction

Thermoelectric half-Heusler (HH) semiconductors, which can realize the conversion from waste heat to electric power through the Seebeck effect, have been widely studied from the viewpoint of increasing demands for sustainable and renewable energy due to the decline of fossil fuel reserves [1,2,3,4]. The performance of thermoelectric materials is described by the dimensionless figure of *ZT* = *S*^2^*σT*/*κ* [5,6]. In the formula, *S*, *σ*, *T*, and *κ* denote the Seebeck coefficient, the electrical conductivity, the absolute temperature, and the thermal conductivity, respectively. One can artificially improve *ZT* by maximizing the power factor [7,8,9,10,11] and minimizing the thermal conductivity [12,13,14,15,16]. Limited by the conflicts among these transport coefficients (*S*, *σ*, and *κ*), searching for materials with an intrinsically high power factor is considered as an effective strategy. Some ternary half-Heusler semiconductors are considered to be potentially thermoelectric materials precisely because of their large power factor, owing largely to the narrow energy bandgap around the Fermi level in the band structure and their superior temperature stability and mechanical properties [17,18,19]. The good thermoelectric properties of half-Heuslers have been verified [20,21]. A new half-Heusler compound, ZrNiPb, was synthesized by Zunger et al. and was found to have a power factor of 5.2 µWcm^−2^K^−1^ and a thermopower of −153.9 µV/K when ZrNiPb was placed in room temperature [22], so ZrNiPb may become a promising crystal for efficient thermoelectric materials. Based on first-principles calculation, the electronic structure and thermoelectric properties of the half-Heuslers ABPb (A = Hf, Zr; B = Ni, Pd) were recently studied [23]. It was found that the four semiconductors have a narrow bandgap, and the calculated power factor firstly increases to a peak and then decreases as the carrier concentration increased. The thermoelectric figure of merit *ZT* of 0.3 can be attained at a high temperature by choosing an appropriate doping level. It is possible to make the half-Heusler ZrNiPb become a potential parent candidate for efficient thermoelectricity by reducing lattice thermal conductivity through point defects and boundaries [24]. Other theoretical and experimental studies [25,26] have also demonstrated that ZrNiPb-based half-Heusler semiconductors are potential parent candidates for high-performance thermoelectric devices.

The narrow bandgaps of half-Heusler alloys are sensitive to pressure, which may influence their thermoelectric behavior [22,27]. Semiconducting half-Heusler alloys have shown to exhibit large Seebeck coefficients at room temperature and moderate electrical conductivities, which are attributed to their narrow bands associated with high effective masses. High effective masses are beneficial for high performance in thermoelectric materials. The elastic modulus is essential to offer a more in-depth understanding of the mechanical properties and hardness of the crystal. On the other hand, the thermodynamic properties are considered to be important parameters to evaluate specific behaviors when the crystals are in a high-temperature or high-pressure environment. Based on the fact that palladium belongs to the same group as nickel, studies on the electronic structures of ZrPdPb may offer useful information for the prediction of their thermoelectric behavior. However, the available information on the electronic properties of ZrPdPb under high pressure is still very limited both in experiment and theory. Predictions of the thermoelectric properties of half-Heuslers are based on reliable calculations of the band structures. In particular, band structures under pressure are very important to determine the response of the crystal to external forces. Additionally, the thermoelectric behavior of real-world thermoelectric devices may be influenced. Therefore, in this paper, the influence of pressure on the dynamic, elastic, and electronic properties of the half-Heuslers ZrNiPb and ZrPdPb are studied, and their thermodynamic properties, like the normalized volume *V*/*V*_0_, heat capacity *C*_v_, and Debye temperature *Θ*_D_, are estimated by minimizing the Gibbs free energy to the volume based on the quasi-harmonic Debye model [28]. We hope this investigation will offer useful guidelines for further understanding of the thermoelectric properties of the half-Heusler semiconductors ZrNiPb and ZrPdPb.

## 2. Computational Details

The half-Heusler semiconductors ZrNiPb and ZrPdPb with the space group of F-43m (No.216) crystallize in a cubic structure [29] consisting of four interpenetrating fcc sublattices. In Wyckoff coordinates, Pb and Zr atoms are located at the A(0,0,0) and C(1/2,1/2,1/2) sites, and the B(1/4,1/4,1/4) site is occupied by Ni or Pd atoms. Otherwise, the D(3/4,3/4,3/4) site is unoccupied. In this work, all of the calculations were performed according to the density functional theory (DFT), as conducted within the Cambridge Sequential Total Energy Package (CASTEP) [30]. The exchange-correlation potential is described by the generalized gradient approximation (GGA) in the Perdew–Burke–Ernzerh (PBE) form [31]. The valence electron configurations of Zr(4d^2^5s^2^), Ni(3d^8^4s^2^), Pd(4d^10^), and Pb(6s^2^6p^2^) were selected, respectively. The Broyden–Fletcher–Goldfarb–Shanno (BFGS) minimization was used to deal with the structural optimizations and electronic structure at different external pressures [32]. After the convergence tests, the kinetic energy cutoff for the plane-wave basis was 540 eV. The Brillouin zone was sampled with a 19 × 19 × 19 Monkhorst-Pack k-point [33]. The atom position was fully relaxed until the energy change per atom was less than 5.0 × 10^−8^ eV, the forces on atoms were less than 0.01 eV/Å, and all stress components were less than 0.02 GPa.

## 3. Results and Discussions

### 3.1. Structural Stability and Dynamic Stability

**Figure 1:**
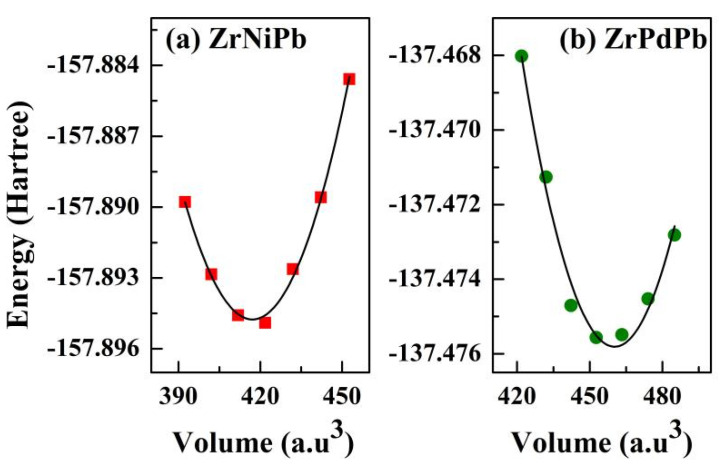
The calculated *E-V* data of ZrNiPb and ZrPdPb primitive cell.

The obtained equilibrium lattice parameters for ZrNiPb and ZrPdPb in ground-state structures are, respectively, 6.252 and 6.485 Å, as shown in Table 1. With the purpose of obtaining the thermodynamic properties of ZrNiPb and ZrPdPb, we further calculated the total energy of ZrNiPb and ZrPdPb as the variation in the lattice constant relative to the equilibrium lattice constant ranging from −0.08 to 0.08, and the plot is shown in Figure 1. By fitting the corresponding energy–volume (*E*-*V*) data of ZrNiPb and ZrPdPb to the third-order EOS formula [34,35], the obtained bulk modulus *B*_0_ (GPa) and its first-order derivative to pressure *B′*_0_ were obtained and are listed in Table 1. It can be clearly noted that our calculated lattice parameters match well with other calculated results [23], indicating that the calculations in this study are highly reliable.

**Figure 2:**
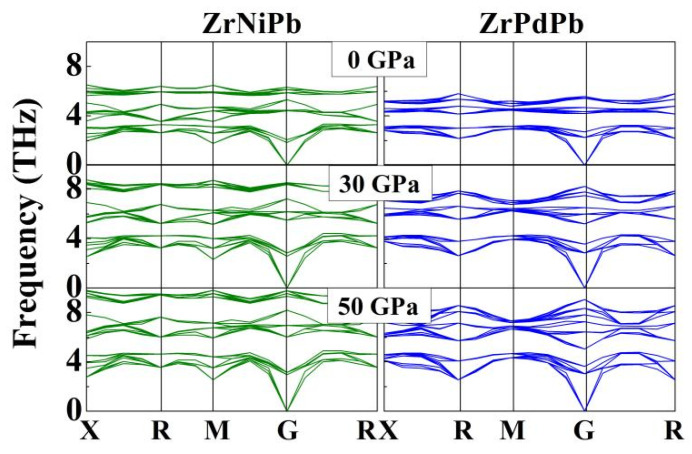
The calculated phonon dispersion curves of ZrNiPb and ZrPdPb at different pressures. The X, R, M, and G represent the highly symmetric points.

After obtaining the ground-state structures of ZrNiPb and ZrPdPb, we further checked their dynamical stability under pressure by calculating the phonon spectra dispersions, as the soft phonon modes reveal the distortion of the crystal. Figure 2 presents the calculated phonon dispersion curves in the whole Brillouin zone of the half-Heuslers ZrNiPb and ZrPdPb at high pressures up to 50 GPa. The inexistence of soft frequencies in all phonon branches confirms the dynamic stability of the half-Heuslers ZrNiPb and ZrPdPb until the pressure increases to 50 GPa. In addition, it was found that the phonon dispersions both in ZrNiPb and ZrPdPb become more and more dispersive as the pressure increases, leading to a higher group velocity and, thus, an enhanced lattice thermal conductivity [36,37,38].

### 3.2. Mechanical Stability and Elastic Properties

**Figure 3:**
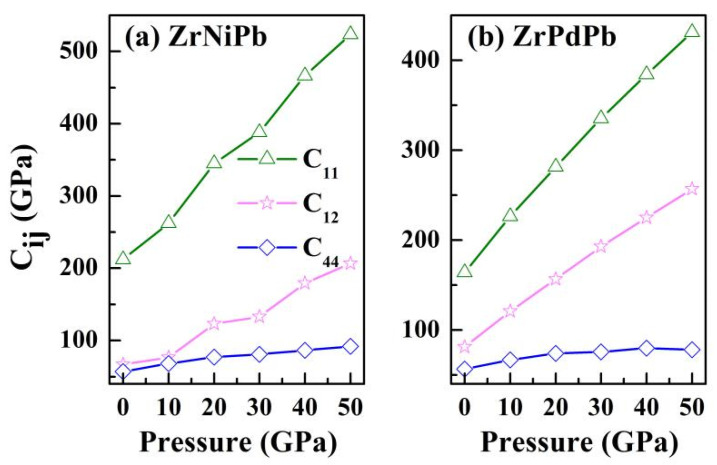
The calculated elastic constants *C*_ij_ versus pressure for cubic ZrNiPb and ZrPdPb.

The ability of a material to recover from stress and deformation is characterized by elastic constants. As the half-Heuslers ZrNiPb and ZrPdPb crystallize in cubic symmetry, there are only three independent elastic constants, namely, *C*_11_, *C*_12_, and *C*_44_ [39]. The three elastic constants, respectively, characterize the longitudinal deformation, transverse expansion, and shear deformation of a material. By computing the stress generated by applying a small strain to an optimized unit cell, the three elastic constants of ZrNiPb and ZrPdPb as a function of pressure were calculated, and the results are illustrated in Figure 3. The mechanical stability of the two half-Heusler alloys is judged by the mechanical stability criteria [40,41]. For ZrNiPb and ZrPdPb, the three elastic constants satisfy these stability conditions, indicating that cubic ZrNiPb and ZrPdPb are mechanically stable. On the other hand, it can be seen that the three elastic constants keep on increasing moderately at all applied pressures. However, the sensitivity of *C*_44_ to increasing pressure is less than that of *C*_11_ and *C*_12_ for both ZrNiPb and ZrPdPb. This indicates that uniaxial deformation is more easily influenced by pressure than shear deformation. The influence of pressure on elastic constants can also be observed in cubic Mn_2_CoAl [42] and Co_2_MnSi [43], for they all belong to Heusler alloys and have the same half-metallicity.

**Figure 4:**
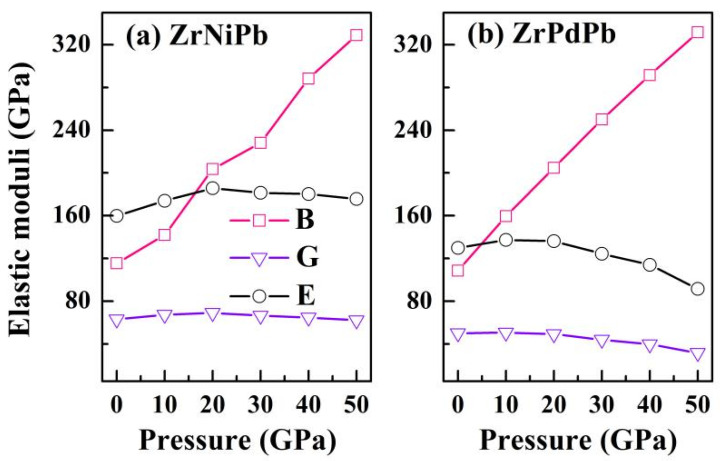
The obtained bulk modulus *B*, shear modulus *G*, and Young’s modulus *E* versus pressure.

After examining the mechanical stability of the half-Heuslers ZrNiPb and ZrPdPb under pressure, we further confirmed other important elastic properties reflected by the bulk modulus *B*, shear modulus *G*, and Young’s modulus *E* based on elastic constants according to the VRH approximation [44]. All elastic moduli of ZrNiPb and ZrPdPb as the pressure increases from 0 to 50 GPa are plotted in Figure 4. It is worth mentioning that the values of *B* from the VRH approximation are 115.254 GPa for ZrNiPb and 108.569 GPa for ZrPdPb, and they have nearly the same values as those obtained from energy minimization, thus indicating the reliability of the method in our calculations. As seen in Figure 4, the bulk modulus monotonically increases as the pressure increases to 50 GPa, showing that it becomes harder and harder to compress cubic ZrNiPb and ZrPdPb. On the contrary, the Young’s modulus values of ZrNiPb and ZrPdPb show a downward trend when the pressure increases beyond 20 GPa. So, ZrNiPb and ZrPdPb become stiffer as the pressure increases from 0 to 20 GPa. When the pressure continues to increase, the stiffness of ZrNiPb and ZrPdPb decreases. The brittle or ductile behavior of a material is determined by the ratio between the bulk and shear moduli (*B*/*G*). A ductile material has a *B*/*G* value greater than 1.75; otherwise, the material is more brittle. In this study, the *B*/*G* values of ZrNiPb and ZrPdPb are larger than 1.75 and increase with increasing pressure. As a result, the ductility of both ZrNiPb and ZrPdPb becomes better as the pressure increases. Moreover, the larger bulk modulus, Young’s modulus, and shear modulus of ZrNiPb suggest its greater hardness, stiffness, and resistance to shear deformation than ZrPdPb at the same pressure. This may be attributed to the smaller atomic mass and cell volume of Ni compared to Pd.

### 3.3. Electronic Properties

**Figure 5:**
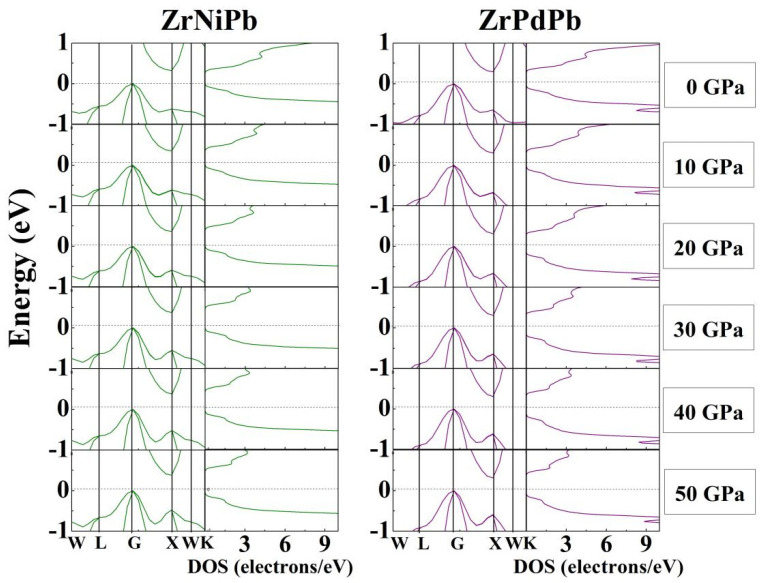
The band structure and density of states as functions of pressure for ZrNiPb and ZrPdPb, respectively. The horizontal dash lines indicate the Fermi level.

The half-Heusler semiconductors ZrNiPb and ZrPdPb are regarded as excellent thermoelectric materials, owing largely to their appropriate bandgaps. The band structure of the thermoelectric materials near the Fermi level provides very useful information to estimate transport properties. To explore their electronic properties under pressure, the energy band structures and densities of states of the half-Heuslers ZrNiPb and ZrPdPb around the Fermi level were calculated and are presented in Figure 5. The left and right parts of both columns are, respectively, the band structures and densities of states of ZrNiPb and ZrPdPb. It can be seen from the band structures that the valence band maximum (VBM) sits at the highly symmetric G point, while the conduction band minimum (CBM) is located at the X point, thus showing that ZrNiPb and ZrPdPb are both indirect-gap semiconductors. The calculated indirect bandgap in ZrNiPb is 0.36 eV, which agrees well with the other theoretical value (about 0.37 eV) obtained by using GGA and GGA + SOC [20]. ZrPdPb exhibits an indirect bandgap of 0.49 eV. Our calculated values of the bandgaps are similar to other experimental and theoretical results [22,23], indicating the reliability of the calculations in this study. Such small values of energy bandgaps are considered to be suitable for moderate temperature thermoelectric applications [45]. As ZrNiPb and ZrPdPb are compressed, the minimum of the conduction band slowly shifts toward higher energies, and the maximum of the valence band remains nearly unvaried when the applied pressure increases to 50 GPa. So, the applied pressure has little influence on the valence bands while it has clear effects on the conduction bands. As the pressure increases, on the other hand, it can also be observed that the density states around the minimum of the conduction band become sharper, resulting in an increase in the effective masses of the electrons. It is concluded that the thermoelectric properties may have greater improvements in the n-type ZrNiPb and ZrPdPb rather than in the p-type ones under pressure. ZrNiPb and ZrPdPb are anticipated to be good moderate-temperature thermoelectric materials under high pressure. When the pressure is zero, the density states around both the CBM and VBM are sharper than those of the Ni-based half-Heuslers NiVZ (Z = Al, Ga, and In) and NiTiZ (Z = Si, Ge, and Sn) [46], indicating the better thermoelectric performance of ZrNiPb and ZrPdPb. As discussed above, more dispersive phonon dispersions both in ZrNiPb and ZrPdPb result in a larger lattice thermal conductivity, which is disadvantageous for obtaining a higher thermoelectric figure of merit. So, it is necessary to select an appropriate pressure to maximize effective masses and minimize thermal conductivity. The half-Heusler semiconductors ZrNiPb and ZrPdPb are also dynamically and mechanically stable up to a pressure of 50 GPa. The thermodynamic properties of ZrNiPb and ZrPdPb are later discussed at a pressure range of 0–50 GPa.

**Figure 6:**
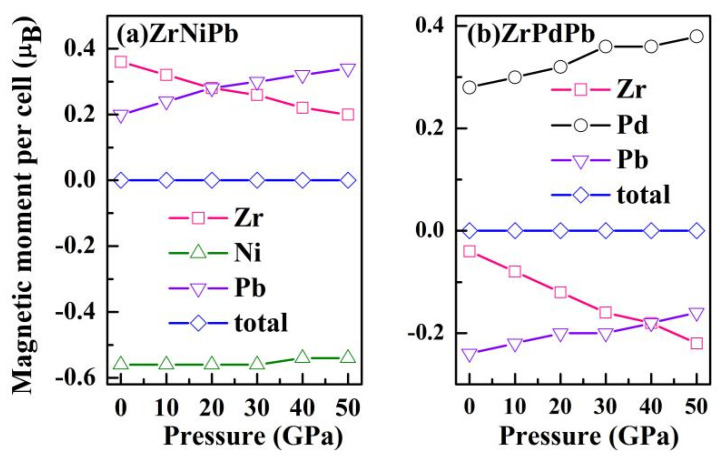
The total and partial magnetic moments of ZrNiPb and ZrPdPb as a function of pressure.

Figure 6 shows the total magnetic moments (TMMs) of ZrNiPb and ZrPdPb and the related atom-resolved moments (AMMs) of Zr, Ni, Pd, and Pb as a function of pressure. It can be seen that both the AMMs of Zr and Pb atoms are positive in ZrNiPb and negative in ZrPdPb. The AMM of Zr in both ZrNiPb and ZrPdPb decreases rapidly as the pressure increases, while that of Pb shows the opposite trend. In addition, the AMMs of Ni in ZrNiPb and Pd in ZrPdPb both exhibit an increasing trend as the pressure increases, while Ni and Pd, respectively, hold negative and positive magnetic moments. Thus, Zr and Pb atoms exhibit antiparallel coupling with the Ni atom in ZrNiPb and with the Pd atom in ZrPdPb. Both ZrNiPb and ZrPdPb hold a TMM of 0 *µ*_B_, and the value remains unvaried at different pressures. In the crystal structure of ZrNiPb (ZrPdPb), it can be seen that Zr and Ni(Pd) are the nearest neighbors. As a result, their d orbitals firstly hybridize, thus creating five new bonding orbitals and five new antibonding ones. Either bonding or antibonding orbitals contain three triple-degenerate t_2g_ and two double-degenerate e_g_. Among them, three t_2g_ orbitals, together with two e_g_ orbitals, are unoccupied, while the others are occupied. Furthermore, the Pb atom provides four orbitals, which can accommodate electrons. Therefore, each unit cell of the half-Heuslers ZrNiPb and ZrPdPb has nine occupied orbitals in total, and the maximum number of electrons is 18. Hence, the SP rule can be described as *M*_t_ = *Z*_t_ − 18. The valence electron numbers of the Zr, Ni(Pd), and Pb atoms are 4, 10, and 4, respectively, resulting in a total electron number, *Z*_t_, of 18 in ZrNiPb and ZrPdPb. So, the total magnetic moments of the half-Heuslers ZrNiPb and ZrPdPb remain zero. Heusler alloys with a zero value for their total spin magnetic moment which are made of magnetic constituents, and which belong to a special class of half-metallic antiferromagnets, are also known as fully compensated ferrimagnets. Half-metallic magnets present metallic behavior in one spin channel, while there exists a gap for the other spin channel, thus creating a 100% spin polarization current in devices. Therefore, the high spin polarization of ZrNiPb and ZrPdPb indicates that they are ideal materials for spin Seebeck investigations and for spintronics in general.

### 3.4. Thermodynamic Properties

**Figure 7:**
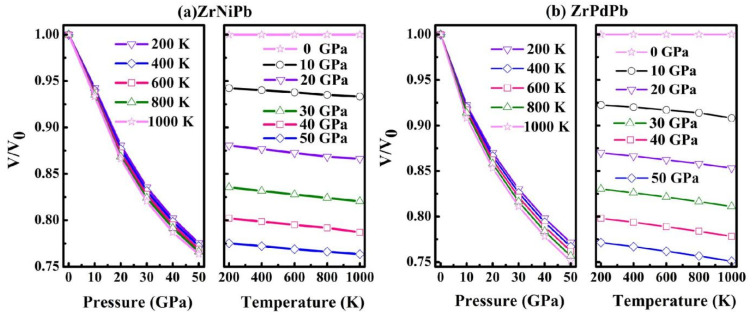
*V*/*V*_0_ as a function of pressure and temperature.

The thermodynamic properties of materials help us to understand their specific behaviors when they are in a high-temperature or high-pressure environment. Therefore, we further explore the heat capacity *C*_v_ and Debye temperature *Θ*_D_ of the half-Heuslers ZrNiPb and ZrPdPb under high temperature and high pressure using the quasi-harmonic Debye model based on *E*-*V* data from the above DFT calculations. The dependencies of the calculated relative volume *V*/*V*_0_ of ZrNiPb and ZrPdPb on temperature and pressure are plotted in Figure 7. It is shown that the relative volume of ZrNiPb and ZrPdPb monotonically decreases as the pressure or the temperature increases. Moreover, the sensitivity of the relative volume to pressure is stronger than that to temperature for both ZrNiPb and ZrPdPb.

**Figure 8:**
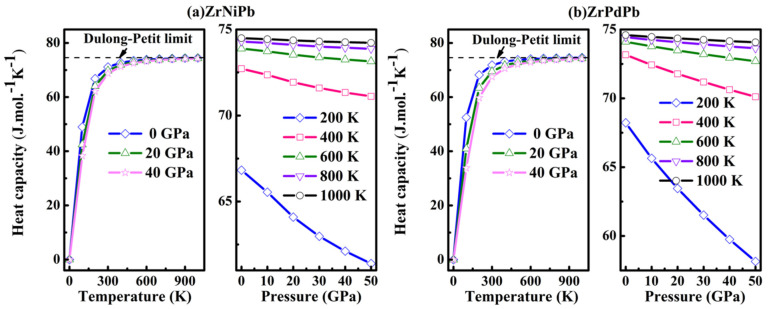
The heat capacity *C*_v_ as a function of pressure and temperature.

Studying the heat capacity *C*_v_ is important to extend our knowledge about lattice vibrations, the energy band structure, the density of state, the transition of phase of the solid, and so on. The pressure and temperature effects on the heat capacity *C*_v_ of ZrNiPb and ZrPdPb are depicted in Figure 8. When the temperature is below 600 K, *C*_v_ is proportional to *T*^3^ at different pressures, obeying Debye’s law. At higher temperatures, the heat capacity *C*_v_ approaches the Dulong–Petit limit. The limit in this work is 74.32 for ZrNiPb and 74.53 Jmol^−1^K^−1^ for ZrPdPb at 0 GPa and 900 K, similar to the 73.97 Jmol^−1^K^−1^ value of NiVSb [47] and the 74.44 Jmol^−1^K^−1^ value of NaCdP [48], which also belong to half-Heusler alloys. As the *C*_v_ value of ZrNiPb is larger than that of ZrPdPb at the same pressure and temperature, we show that the ability of absorbing the heat of ZrNiPb is stronger, and it is thermodynamically more stable than ZrPdPb [48]. These calculative values of heat capacity *C*_v_ for ZrNiPb and ZrPdPb are helpful to give guidelines for other theoretical and experimental works.

**Figure 9:**
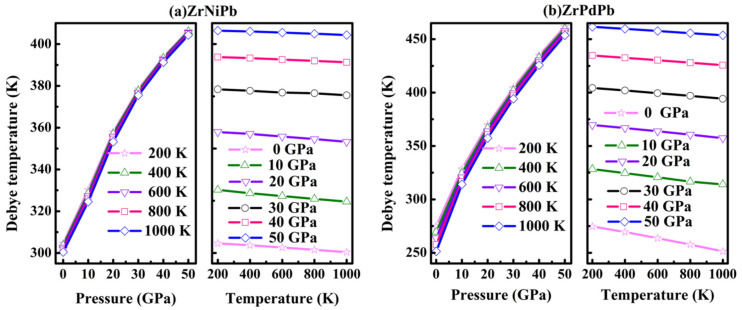
The Debye temperature *Θ*_D_ as a function of pressure and temperature.

In addition, the Debye temperature *Θ*_D_ is another physical parameter of a crystal, which can provide information about specific heat, elastic constants, vibrations of lattice, and the melting temperature. When the temperature is below the Debye temperature, the vibrational excitations of the lattice occur mainly from acoustic vibrations, and the quantum mechanical effect makes an important contribution to tune the thermodynamic properties. Otherwise, it can be neglected when the temperature is above the Debye temperature. The variation of *Θ*_D_ as a function of pressure and temperature for ZrNiPb and ZrPdPb is plotted in Figure 9. It is demonstrated that *Θ*_D_ increases almost linearly as the pressure increases at a given temperature. It can also be seen that *Θ*_D_ shows a downward trend with an increasing temperature.

## 4. Conclusions

The elastic, electronic, and thermodynamic properties of the half-Heuslers ZrNiPb and ZrPdPb under pressure were studied by employing first-principles calculations and the quasi-harmonic Debye model. Our calculated equilibrium lattice parameters and semiconducting behaviors are consistent with other theoretical results. The dynamic stability of ZrNiPb and ZrPdPb under pressure was confirmed by calculating the phonon spectra dispersions. The elastic constants under the pressure range of 0–50 GPa satisfy the generalized elastic stability criteria, indicating the mechanical stability of cubic ZrNiPb and ZrPdPb. According to VRH approximation, the obtained elastic constants indicate that the ductility of the two compounds becomes better as the pressure increases. We further reported the influence of pressure on the electronic structures of ZrNiPb and ZrPdPb and found that the energy bandgap keeps increasing as the pressure rises to 50 GPa. Furthermore, the thermodynamic properties under different pressures and temperatures were calculated. The calculated Dulong–Petit limit was 74.32 for ZrNiPb and 74.53 Jmol^−1^K^−1^ for ZrPdPb at 0 GPa and 900 K. We hope these results will provide guidelines for further theoretical and experimental works.

## Figures and Tables

**Table 1:** The calculated equilibrium lattice constants *a*_0_ (Å), bulk modulus *B*_0_, and its pressure derivative *B′*_0_ (GPa) in comparison with the available values.

Compound	*a*_0_	*B*_0_	*B′*_0_
ZrNiPb	6.252	117.35	4.30
Cal. [23]	6.267		
ZrPdPb	6.485	106.40	4.96
Cal. [23]	6.506		

## Data Availability

Data are contained within the article.
